# A Continued Form of Remittent Fever of La Grippe

**Published:** 1892-10

**Authors:** J. Samuel Price

**Affiliations:** County Physician, Beaumont


For Daniel's Texas Medical Journal.

### A CONTINUED FORM OF REMITTENT FEVER OF LA GRIPPE.

J. SAMUEL PRICE, M. D., COUNTY PHYSICIAN, BEAUMONT.

Read before the Southeast Texas Medical Society, September 6, 1892.

UPON reviewing the literature recently published bearing on the sequelæ of la grippe, we are struck with the similitude existing with the remote effects of the late epidemic and that of malaria; in fact, so protean are its manifestations that the writer of this paper fails to see wherein the Society could become interested in a resume of some of the obscure effects which a few of our learned friends ascribe to la grippe; hence we will confine ourselves to the consideration of the most frequent sequela. Some have ventured so far as to claim to be able to trace a connection between affections originating two or three years subsequent to the attack, and it would appear to a casual observer, if this fact were tenable, that we might ascribe some of the alleged blunders of the present administration to remote sequences of this protean pandemic. Yet, we cannot but believe that there are morbid processes, perhaps lying dormant, which become lighted by the depressing effects of la grippe, and we are equally convinced that existing effections become very much aggravated by the inception of influenza.

We have become very much interested in observing the primary effect upon the nervous system, which, not unlike other depressing agencies, gives rise to certain unmistakable symptoms. It was our pleasure to attend, in company with Dr. Elliott, several cases, besides those not in our own practice, of protracted fever during the prevalence of the late epidemic, which we believe due to the depression of certain centres in the nervous system, of which we will have more to say later. One of our medical friends rather opposed this theory, by reason of having come in contact, during his practice here, with a continued form of fever in every way analogous to that observed in quite a number of our patients.

But we must remember that fever, like the chill that usually precedes it, is not wont to take on any peculiarities that would furnish us with an index to the etiological factor producing it. Any agent that will sufficiently depress the vaso-motor centres, will be followed by chill, let it be malaria, pneumonia, meningitis, septicemia, or la grippe; likewise any agent that will produce a corresponding depression of the trophic centres will be followed by fever of a varying duration, dependent on the presence of the cause.

These latter centres have been found to preside over the energy expended in tissue building, and any agent depressing them causes that energy to be directed from raising pabulum into tissue, into the next lower order of energy, that of the production of fever, or an excess of the normal temperature.

Prof. John B. Elliott says: "In a healthy person who is invaded by fever, we find, first, a state of nervous failure; the approach of this is heralded by some days of malaise. When finally the nervous system can no longer contend against the depressing cause, a sudden and marked nervous prostration ensues; this is the period of chill, during which the great mass of blood gravitates through capillary relaxation to the great abdominal viscera. This condition of chill is a phenomenon as general almost as that of fever. It belongs to no specific disease but is the expression of nervous failure that generally precedes the subsequent febrile state. Even in this condition of chill, however, increased temperature is revealed by the thermometer. The same condition of nervous disturbance that will account for the increased temperature of true fever, is here already in existence, for we must truly regard even the sthenic febrile condition as still one of nervous depression—a continuation of the state so suddenly announced by the chill. The cause of this increased heat production seems, from what has been said, to be capable of explanation as follows: In a healthy body at rest the transformation of chemical energy goes on under the control of the nervous centres in two principal directions; first, a certain amount is transformed into heat, by which the normal body temperature is maintained; secondly, the remainder is transformed, as has been taught by LaCote, into that power that lifts pabulum into tissue. This tissue-building force represents a certain amount of work done, and for it we are compelled to seek some constant origin in the process of force-transformation that goes on in the body. The transformation of chemical energy is the only such origin known to us. If these almost self-evident propositions are granted, then the explanation of the phenomenon of fever is simple.

Thus, some agent, whether through the blood or directly, disturbs the normal balance of the nervous system. Its most important, and probably the highest physical functions, tissue-building, is arrested; combustion, nevertheless, goes on, but now that portion of chemical energy wont to be transformed into tissue-building force, must seek another outlet, and take the lower form of transformation into heat. In other words, chemical energy, which, under the control of the nervous system, was, in health, being transformed in two directions, tissue-building force, and heat, now, upon the failure of the nervous system, is transformed only in one direction, heat. But more than this must be supposed. During the febrile condition the tissues of the body not only cease in a measure to be renewed, but those already formed seemed to disintegrate more rapidly. With the destruction of that power in the nervous system which raises pabulum to tissue, there also seems to fail a power maintaining tissue integrity. The process of emaciation, as well as the great increase of albuminoid excreta observed during fever, demonstrates this.

We must therefore define fever in terms of the nervous system. Fever must be defined as a pathological condition, resulting from a failure of the nervouse centrès controlling tissue repair and tissue integrity, during which the transformation of chemical energy into tissue-building ceases, and its transformation into heat ensues; and we must add during which, through the same nervous failure, tissue already become built becomes readily subject to combustion.

If we will observe the following cases occuring in one family, we might ascribe a degree of contagiousness to la grippe, a theory advanced by a recent writer in the last edition of the Annual of the Universal Medical Sciences. The first case, a gentleman aged thirty-five, by occupation a sawmill laborer, was stricken down with influenza, the usual symptoms of the nervous type predominating; fever came on gradually without any initial chill, registering only about 100 in the morning with a rise of a half degree in the afternoon, never being entirely clear of fever through the night. Fever gradually increasing to 105 in the third week. Headache of frontal region very distressing without any intermission during the first two weeks. Slight aberration of the mind ensued after first week.

Persisent insomnia up to the second week, accompanied by extreme restlestness. Bowels sluggish, appetite absent. Pulse weak, numbering from 100 to 120. Respiration from about 18 to 24 per minute. Cough though present, not annoying. Duration of fever five weeks. Tedious and protracted convalesence.

The second patient is the wife of the gentleman whose history has been given above. She was taken sick about a week after her husband began to convalesce, symptoms differing somewhat from the preceding, the respiratory tract being more involved, with some disturbance of the bowels. About four months advanced in pregnancy, but no symptons of miscarriage supervened. Fever of remittent type with evening exacerbation, reaching as high as 100 in the third week. Insomnia present, though not so persistent as encountered in previous case. Examination of the lungs revealed a few mucus rales on both sides. At one time streaks of blood were noticed in sputa; never any dullness on percussion. Cough gradually subsided towards last of second week, bnt fever continued unabated.

After the expiration of about a week from the time the lady began to convalesce one of her children, a girl about ten years of age, sickened with the influenza and with symptoms very much like these observed in the former histories. The duration of her fever was about four weeks.

For fear of tiring the members of the society with long and tedious histories, we will conclude our remarks by refering to a paper written by Dr. John H. Hollister, of Chicago, Ill., entitled "A Modified Form of Continued Fever Following the Epidemic Grippe," published in the *Medical Record* June 11, 1892, in which he says: "This disease has seemed to increase the severity of all our endemic maladies." He had observed following the epidemic many cases of continued fever which had been admitted to hospital classified as typhoid. He soon began to doubt the corectness of the diagnosis. A study of fifty cases had afforded the following data: No common cause could be assigned. Previous history was negative. Some had had grippe, but the majority had not.

The prodroma lasted four days, with extreme muscular soreness. The onset was gradual, no chill being noticed, but the fever was continuous without intermission. The average duration was twenty-three days, relapses common, but could be attributed in many cases to dietetic errors. There were no head symptoms nor coma and no subsultus. In four cases there was profuse sweating, lasting over ten days.

The stomach was not troublesome. Secretions were all diminished, and there were no critical discharges.

The mouth was rarely dry, tongue not fissured, and no soreness. The dorsum was milk white. There was no tympanites nor abdominal tenderness; no peritonites, while the bowels were bound up.

In only three cases did the stools indicate typhoid. No bacteria were found in the stools. The urine did not respond to the Ehrlich test, nor were any albumin, sugar, or casts found. There was no rash. In treating the cases main reliance was placed on sponging and packs. Three cases died, one from pneumonia, and two from exhaustion. No intestinal lesions were found at any of the autopsies.

